# Parkinson's disease and vitamins: a focus on vitamin B12

**DOI:** 10.1007/s00702-024-02769-z

**Published:** 2024-04-11

**Authors:** Arwa Rekik, Carlo Santoro, Karolina Poplawska-Domaszewicz, Mubasher Ahmad Qamar, Lucia Batzu, Salvatore Landolfo, Silvia Rota, Cristian Falup-Pecurariu, Iulia Murasan, Kallol Ray Chaudhuri

**Affiliations:** 1 Department of Neurology of Sahloul Hospital, Sousse, Tunisia; 2 Faculty of Medicine of Sousse, Sousse, Tunisia; 3 Department of Basic Medical Sciences, Neurosciences and Sense Organs, University of Bari “Aldo Moro”, Piazza Giulio Cesare 11, 70100 Bari, Italy; 4 Department of Neurology, Poznan University of Medical Sciences, 60-355 Poznan, Poland; 5 Parkinson’s Foundation Center of Excellence, King’s College Hospital, Denmark Hill, London, UK; 6 Division of Neuroscience, Department of Basic & Clinical Neuroscience, Institute of Psychiatry, Psychology & Neuroscience, King’s College London, London, SE5 9RT UK; 7 Faculty of Medicine, Transilvania University of Brasov, 500036 Brasov, Romania; 8 Department of Neurology, County Clinic Hospital, Brasov, Romania

**Keywords:** Parkinson’s disease, Vitamin B12, Vitamins, Homocysteine, Motor, Non-motor

## Abstract

Parkinson’s disease (PD) has been linked to a vast array of vitamins among which vitamin B12 (Vit B12) is the most relevant and often investigated specially in the context of intrajejunal levodopa infusion therapy. Vit B12 deficiency, itself, has been reported to cause acute parkinsonism. Nevertheless, concrete mechanisms through which B12 deficiency interacts with PD in terms of pathophysiology, clinical manifestation and progression remains unclear. Recent studies have suggested that Vit B12 deficiency along with the induced hyperhomocysteinemia are correlated with specific PD phenotypes characterized with early postural instability and falls and more rapid motor progression, cognitive impairment, visual hallucinations and autonomic dysfunction. Specific clinical features such as polyneuropathy have also been linked to Vit B12 deficiency specifically in context of intrajejunal levodopa therapy. In this review, we explore the link between Vit B12 and PD in terms of physiopathology regarding dysfunctional neural pathways, neuropathological processes as well as reviewing the major clinical traits of Vit B12 deficiency in PD and Levodopa-mediated neuropathy. Finally, we provide an overview of the therapeutic effect of Vit B12 supplementation in PD and posit a practical guideline for Vit B12 testing and supplementation.

## Introduction

Parkinson’s disease (PD) is a multicomplex, multi-neurotransmitter, progressive chronic disease which encompasses not only motor features (such as bradykinesia, rigidity, tremor, and gait disturbance) but also nonmotor features (including constipation, neuropathy, pain, and cognitive decline (Sauerbier et al. 2016).The exact pathophysiology of PD is likely multifactorial amongst which oxidative stress, neuroinflammation and mitochondrial dysfunction may play a role in the development and progression of PD. The involvement of vitamins and minerals in the context of PD is not well understood however, vitamins are micronutrients playing a pivotal role in neurogenesis, neurotransmission, and housing antioxidative properties essential in maintaining homeostasis within the body and brain (Kumar et al. 2022; Rai et al. 2021).

Vitamins are classified as either being fat-soluble or water-soluble vitamins. Fat soluble vitamins, vitamins A (Vit A), D (Vit D), E (Vit E), and K (Vit K), mainly bind to cellular nuclear receptors with the ability to affect the expression of certain genes (Chawla and Kvarnberg 2014; Pignolo et al. 2022). Water soluble vitamins, vitamin C (Vit C) and B-complex vitamins including vitamins B6 (Vit B6), B12 (Vit B12), and folate, affect enzymatic activity by acting as cofactors (Chawla and Kvarnberg 2014). Given the importance of vitamins in the function of the human body and its development, vitamin deficiencies can manifest with significant clinical symptoms and syndromes. Specific vitamin deficiencies can particularly manifest as parkinsonism features.

### Vitamins and parkinsonisms: a brief overview

#### Atypical parkinsonisms

**Fig. 1:**
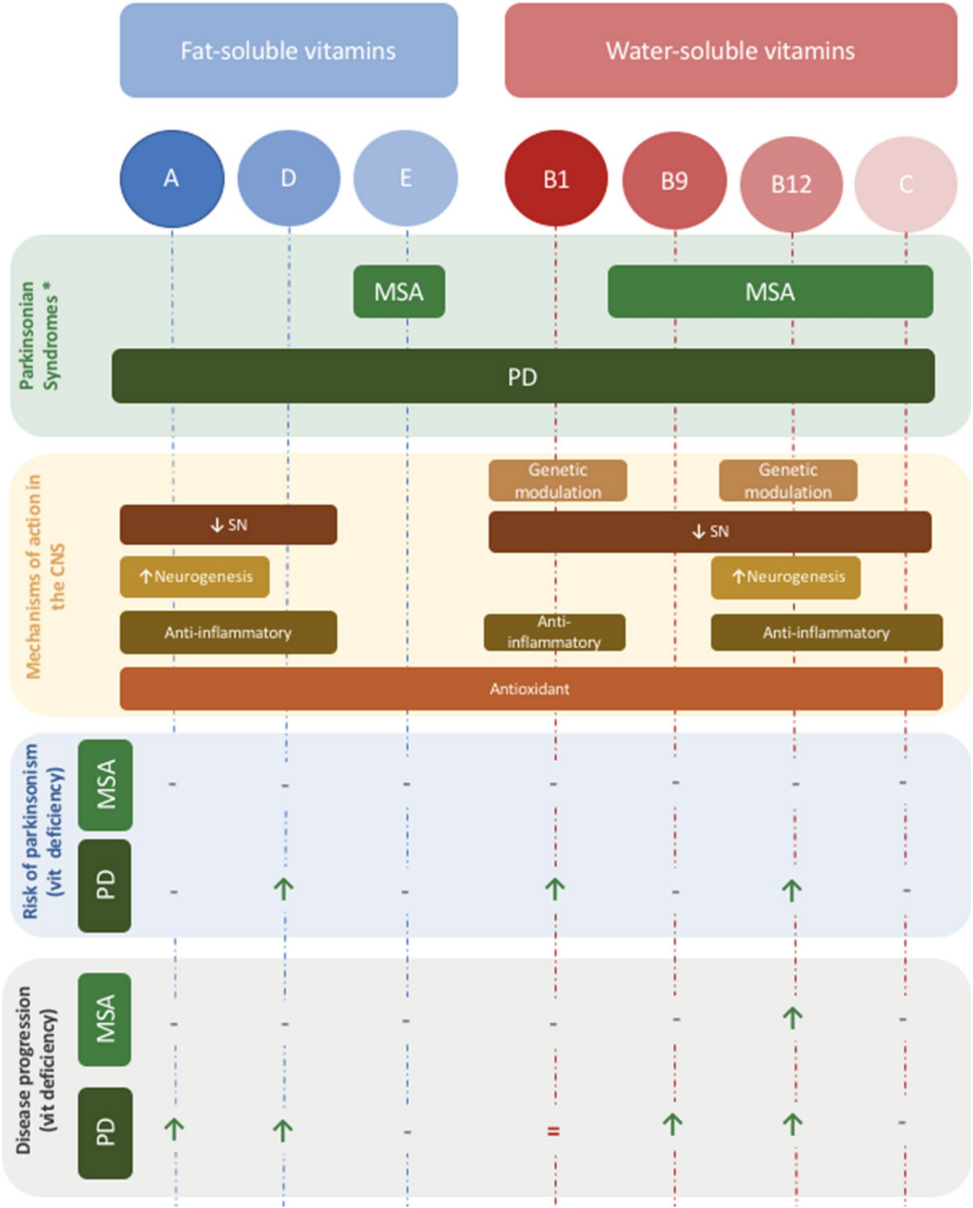
Illustration of the major water-soluble (in red) and fat-soluble vitamins (in blue) linked to neurodegenerative parkinsonian syndromes based on literature review. The green upper section includes the neurodegenerative parkinsonians syndromes cited below specific related vitamins. The orange section indicates the mechanisms of action of each of these vitamins in the CNS in relation with the pathophysiology of parkinsonian syndromes. The blue section points out the vitamin deficiencies associated to higher risk of developing PD and/or MSA (‘***↑****’:*indicates higher risk of PD or MSA in case of the specified vitamin deficiency, ‘- ‘: indicates that the lack of evidence in literature findings. The grey section showcases the association of vitamin deficiency and greater progression in MSA/PD *(‘↑’:* indicates more rapid disease progression in case of the specified vitamin deficiency, ‘ = ’: indicated similar progression rate regarding the presence or not of vitamin deficiency, ‘- ‘: indicates that the relationship between the vitamin deficiency and PD/MSA has not been investigated). *CNS* central nervous system, *PD* Parkinson’s disease, *MSA* multiple system atrophy, *SN* Synuclein, *Vit* vitamin, *Neurodegenerative parkinsonian syndromes

Within the spectrum of neurodegenerative parkinsonian syndromes, multiple system atrophy (MSA) has been notably linked to a significant decrease in Vit B9 levels in a controlled study involving 182 patients (Chen et al. 2022). Since the body cannot synthesize Vit B9, it must be supplemented exogenously and gastrointestinal dysfunction, a prominent dysautonomic feature of MSA, may explain such deficiency. In terms of phenotypic presentation and disease’s progression, low Vit B12 level was correlated with more rapid progression and shorter survival in MSA (McCarter et al. 2020a) **(**Fig. 1**)**.

However, to our knowledge, there is a dearth of data exploring the connection between vitamin deficiencies and corticobasal syndrome (CBS), supranuclear progressive palsy (PSP), and dementia with Lewy bodies (DLB).

#### Vitamins and Parkinson’s disease

In PD, Vit D deficiency has been shown to contribute, not only to the reduction in dopamine levels, but may also be involved in the accumulation of alpha-synuclein (αSN), a hallmark of PD pathophysiology (Zhou et al. 2019). The exact relationship of Vit D and PD remains unclear, given controversial findings with vitamin D supplementation not always impacting the clinical manifestation or the level of disability in PD (Pignolo et al. 2022). Furthermore, several studies reported low vitamin D levels with higher risk of developing PD (Barichella et al. 2022). Vit D levels have shown to consistently be inversely associated with motor symptoms severity, however, this may very well be limited exposure to Vit D due to limited mobility (Fullard and Duda 2020).

Vit A, a fat-soluble vitamin, is characterized with anti-oxidant and anti-inflammatory actions within the brain (Marie et al. 2021). In fact, such mineral acts by reducing the release of pro-inflammatory cytokines and increasing of anti-inflammatory factors. PD is considered as a pro-inflammatory state of the brain mainly due to the abnormal activation of microglia and astrocytes (Fuzzati-Armentero et al. 2019). Thus, vitamin A could reduce dopaminergic cell loss and reduce the underlying neuroinflammation and synuclein aggregation (Ono and Yamada 2007; Takeda et al. 2014). Regarding the clinical implication, low dietary intake of Beta-carotene was associated to greater progression of PD (Takeda et al. 2014; Marie et al. 2021; Yang et al. 2017). However, the association data is indirect and is unlikely to be a robust correlation (Yang et al. 2017) **(**Fig. 1**)**.

Being anti-oxidants, vitamins C and E may theoretically play a role in neuroprotection by decreasing glutamate-mediated excitotoxicity and interfering with αSN oligomerization (Kocot et al. 2017; Martin et al. 2002; Miranda-Díaz et al. 2020). Vitamin C, in particular, solicits dopaminergic neuron differentiation as demonstrated in in-vitro studies (He et al. 2015) and reduces neuroinflammation in MPTP-induced PD animal models (Nuccio et al. 2021).

Vitamin B1 (thiamine) was also linked to PD via genomic mechanisms [including DJ-1 gene mutation (Bonifati et al. 2003)], and non-genomic mechanisms such as oxidative stress and cellular metabolism (Lu’o’ng et al. 2012; Plaitakis and Shashidharan 2000). Thiamine deficiency is associated with a decrease of α-ketoglutarate dehydrogenase complex within the substantia Nigra pars compacta, leading the more severe neurodegeneration (Liu et al. 2017). Furthermore, It has been linked to higher risk of developing PD in one study (Håglin et al. 2017). However, further investigations are needed to elucidate the therapeutic potential of vitamin B_1_ in PD.

Among all vitamins, Vit B12 appears to have the most robust link with PD. To begin with, PD patients are more prone to developing Vit B12 deficiency which may be linked to several factors such as typical low-protein diet in LDopa treated PD, delayed gastric emptying due to autonomic dysfunction, bacterial overgrowth along with helicobacter pylori (HP) infection aggravated by the occurrence of constipation (Fasano et al. 2015). Furthermore, Vit B12 status is more likely to present a potential determinant factor of developing PD. In fact, a recent study investigating the impact of Vit B9, B12 and B6 and the risk of developing PD highlighted that only higher baseline dietary intake of Vit B12 was significantly associated with a lower risk of PD in comparison with the rest of the vitamins (Flores-Torres et al. 2023). In PD, the impact of Vit B12 deficiency can be mediated either by a direct impact of Vit B12 deficiency (Choi et al. 2021; Jia et al. 2019; Luthra et al. 2020; McCarter et al. 2019), or indirectly by the induced hyperhomocysteinemia (HHcy) (Christine et al. 2018; Lau et al. 2006; Periñán et al. 2023; Phokaewvarangkul et al. 2023; Song et al. 2022; Zhang et al. 2015). Vit B12 status is also considered as a modulating factor of the phenotypic expression of PD as recent data have intriguingly suggested a potential role of Vit B12 deficiency in motor progression in PD assessed using the Unified Parkinson’s Disease Rating Scale (UPDRS) and the ambulatory capacity score and the worsening of cognitive dysfunction evaluated via the mini-mental status examination (MMSE) (Christine et al. 2018; Sandeep et al. 2023).

In this pictorial review, we sought to focus on the link between PD and Vit B12 and holistically pinpoint the peculiarities of Vit B12 effect on the molecular, neuropathological and genetic aspects of PD and overview the phenotypic expression, both motor and non-motor, of PD in the light of Vit B12 deficiency. Finally, we aim to outline the key therapeutic approaches in PD implementing the use of supplementation of Vit B12 and its outcomes.

## Methodology

The selection of Vit B12 as the focus of this study was based on its emerging significance in PD pathophysiology and clinical manifestations. Given the vital role of Vit B12 in neural function, its deficiency may contribute to neurodegeneration and exacerbate motor and non-motor symptoms in PD patients. Vit B12 deficiency plays an important role in patients who chronically use oral Levodopa (LDopa) and those who undergo its intestinal formulation, contributing to the acceleration of damage to peripheral nerves. Therefore, a comprehensive exploration of the role of vitamin B12 in PD is warranted to understand its implications for disease management and potential therapeutic interventions.

### Literature search

**A **systematic literature search was conducted using electronic databases including “PubMed” and “Scopus”. The search strategy included combinations of keywords such as "Parkinson's Disease", “Parkinsonism", “Vitamin B12", "Neuropathy", "Homocysteine", "Cognition", "Motor", "Non-motor", "therapeutic", “supplementation”, “treatment” and “outcome”.

### Assessment of vitamin B12 status

The assessment of Vit B12 status in included studies varied with measurements typically conducted through serum or plasma levels of Vit B12. Additional biomarkers such as homocysteine and methylmalonic acid (MMA) were also considered to evaluate functional deficiency. The diagnostic criteria for Vit B12 deficiency followed standard clinical guidelines with levels below 200 pg/mL indicating its deficiency.

### Analysis and interpretation

The extracted data were qualitatively synthesized to identify patterns, trends, and associations between Vitamin B12 status and PD clinical manisfestations. Findings were categorized into motor symptoms (such as gait disturbances and neuropathy), non-motor symptoms (focusing on cognitive decline), pathophysiological mechanisms (based on molecular pathways, neuropathology, metabolites and genes), and therapeutic implications (Vit B12 supplementation, clinical recommendations).

### Clinical recommendations

Based on the synthesized evidence, recommendations for clinical practice were developed. These recommendations aimed to guide healthcare providers managing levodopa–carbidopa intestinal gel (LCIG)–treated patients in the assessment, monitoring, and management of Vit B12 deficiency.

### The pathophysiological links between Vit B12 and PD: complex and robust dynamics

#### Vit B12: from the gut to the brain

Vit B12, also known as cobalamin, is a water-soluble vitamin characterized by a remarkably intricate molecular structure. This structure reflects the complexity of the processes governing the absorption and transportation of this vitamin within the body and into the brain.

The sources of intake of VitB12 include meat, eggs, and dairy products with an average estimated daily consumption equivalent to 2.4 µg per day per person. Vit B12 is mainly bound to food proteins. Thus, it must be liberated to couple with the dedicated transport proteins.

Transcobalamin receptor CD320 can be found in endothelial cells at the blood–brain barrier. It's responsible for taking in and moving Vit B12 into the central nervous system (CNS) (Orozco-Barrios et al. 2009; Wu et al. 2023).

Once into the brain, Vit B12 interacts with genes (LRRK2), proteins (synuclein and Lewy bodies), neurotransmitters (dopamine, Acetylcholine (Ach)) and metabolites that do impact on CNS hemostasis.

#### Vit B12 pathophysiological fingerprints in PD

##### Vit B12 and neural pathways in PD

Vit B12 has been touted as a crucial component of the cholinergic pathway within the brain in several neurodegenerative diseases (El-Mezayen et al. 2022) and recently non-motor subtypes of PD including a cholinergic subtype in particular has been described (Aarsland et al. 2021; Bohnen et al. 2022; Sauerbier et al. 2016). Such vitamin has also been linked to the dopaminergic pathways. In Transcobalamin-Oleodin animal models, Vit B12 deficiency induced dopaminergic caspase-2 mediated cell-death in substantia Nigra (Orozco-Barrios et al. 2009).

**Fig. 2:**
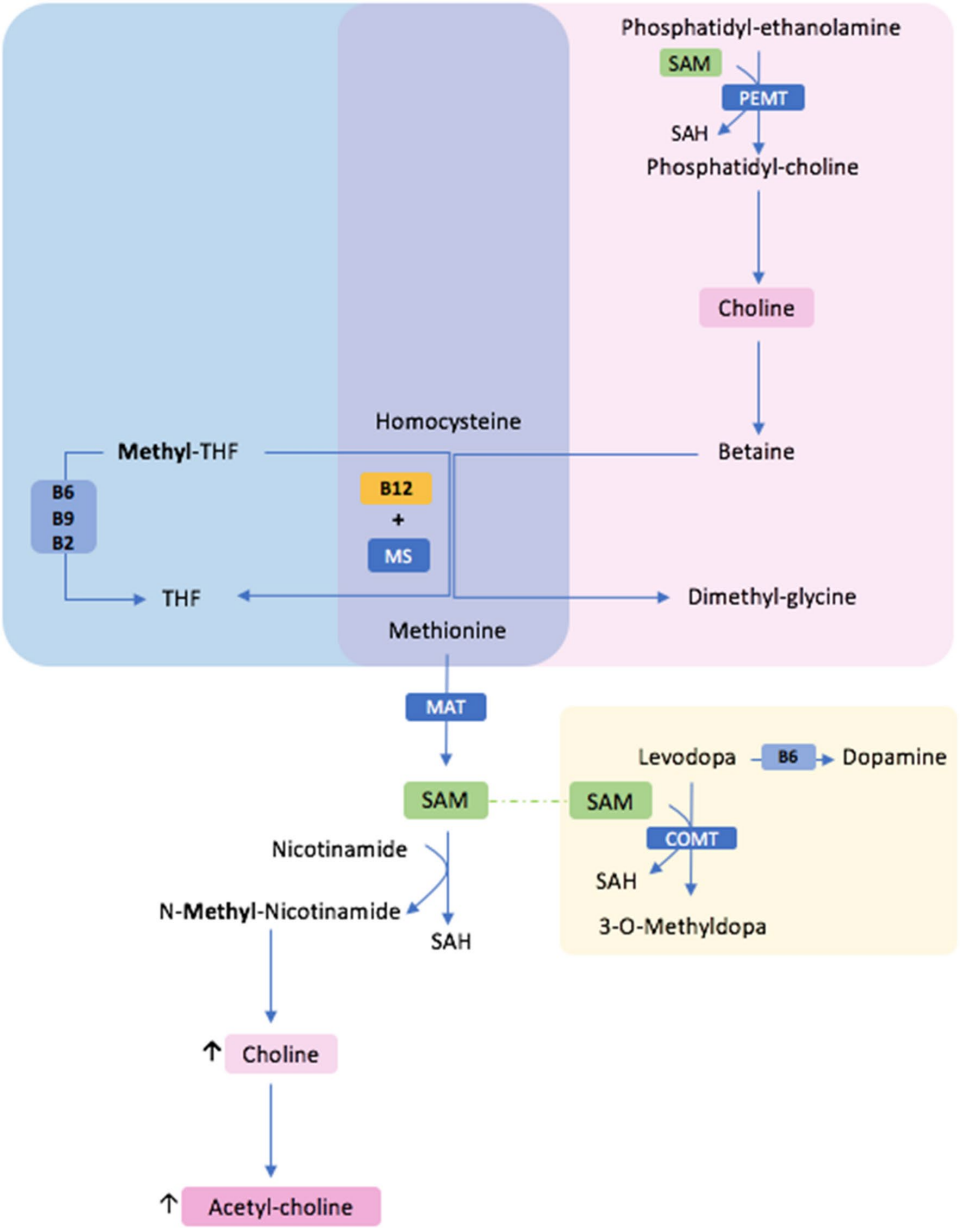
Pictorial illustration of the homocysteine methylation reaction into methionine (in purple) and its link with the cholinergic system. Illustrated in blue is the preferential pathway which is vitamin B-dependent (B12, B6, B9, B9) where the methylation process of homocysteine requires the synergic action of Vit B12 and MS. In Pink, if the secondary methylation pathway that is favored in case of Vit B12 deficiency pinpointing choline transformation to Betaine, the key ‘methyl’ donor for homocysteine. A joint pathway starts with the transformation of methionine into SAM. SAM will serve for the transformation of nicotinamide into N-methy-nicotinamide which inhibits competitively the efflux of choline out of the CNS. In yellow is the pathway of Levodopa transformation into dopamine and into 3-O-Methyl-Dopa. *B6* vitamin B6, *COMT* Catheco-O-Methyl-transferase *MAT* methionine adenosyl-transferase, *MS* Methionine systhetase, *PEMT* Phosphatidyl-ethanolamine N-methyl-transferase, *SAH* S-adenosyl-homocysteine, *SAM* S-adenosyl- methionine, *THF* Tetrahydro-folate

The direct role of Vit B12 is to serve as a co-factor in the methylation reaction of Hcy to methionine acting in synergy with the enzyme: Methionine Synthetase (MS) which would integrate methionine via a series of reactions and serves to produce S-adenosyl-methionine (SAM), a major ‘methyl’ donor for several methylation pathways (Fig. 2). In the scenario of cobalamin deficiency, the methylation of Hcy to methionine will essentially lead to an excessive turnover of Choline resulting in a posited cholinergic deficiency.

#### Vit B12 and synucleinopathy

**Fig. 3:**
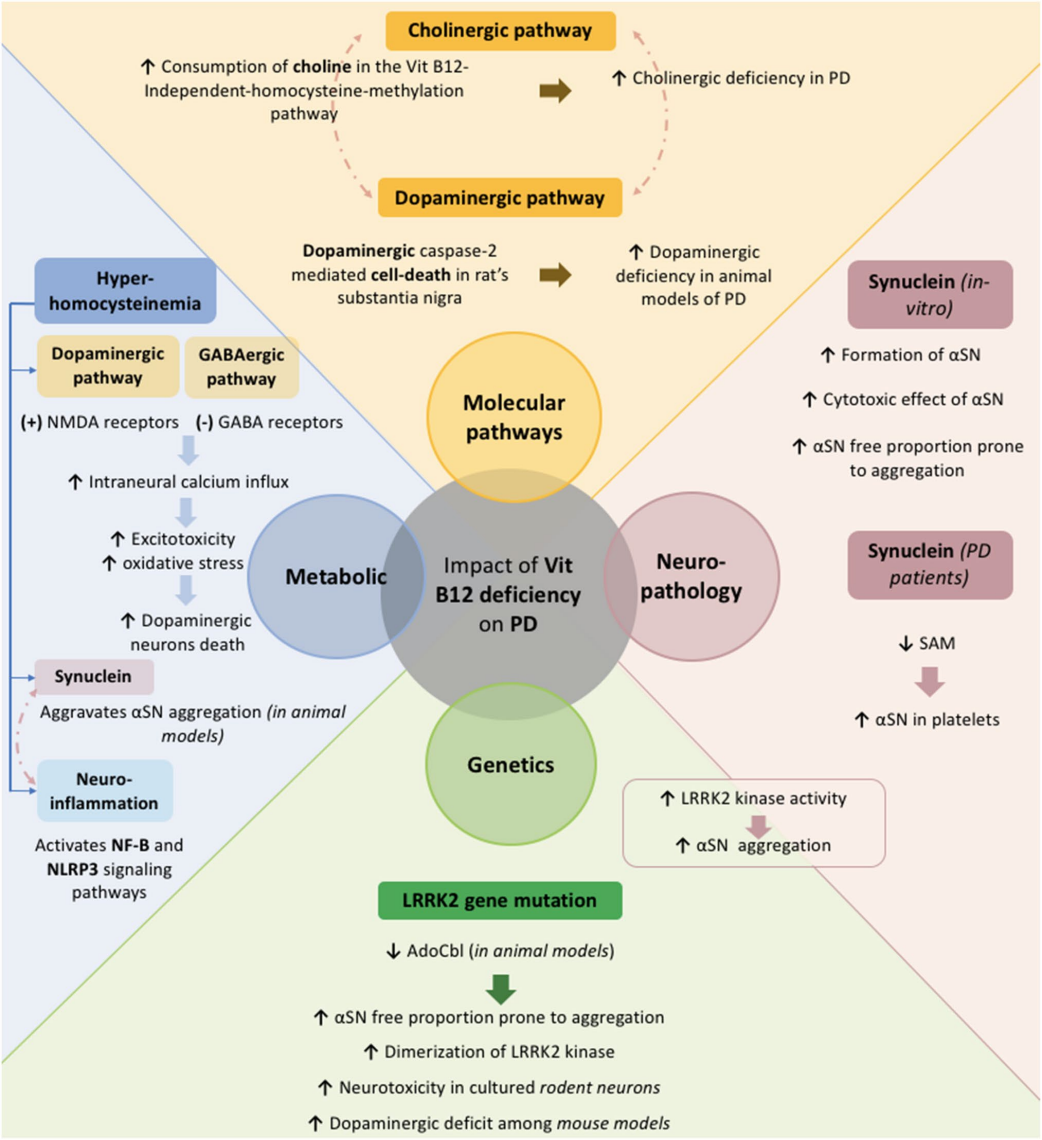
Summary of the different mechanisms of how Vit B12 could potentially interact with the physiopathology of PD. The yellow section focuses on evidence regarding the impact of Vit b12 deficiency on both cholinergic and dopaminergic pathways. The pink section summarizes how Vit B12 deficiency aggravates αSN pathology. Illustrated in the green section Vit B12 deficiency interaction with PD genetic background. In blue, the mechanisms of HHcy induced neurotoxicity in case of Vit B12 deficiency***.*** The dotted pink arrow implies the presence of interaction between the two specified elements. *NLRP-3* Nod-like receptor pyrin 3, *NF-B* nuclear factor kappa B, *LRRK2* Leucine-Rich Repeat Kinase 2, *αSN* alpha-synuclein

A recent study demonstrated that Vit B12 effectively impedes the formation of αSN fibrils in a manner dependent on dosage in-vitro experiments. Circular dichroism spectroscopy data indicated that Vit B12 delays the conformational transition of αSN into β-sheet-rich structures, with a particular influence on the parallel β-sheet conformation. Consequently, Vit B12 significantly alleviated the cytotoxic effects associated with αSN aggregates. Furthermore, it exhibited the ability to bind directly to αSN and to dismantle preexisting mature αSN fibrils and alleviate the ensuing cytotoxicity (Jia et al. 2019) (Fig. 3).

To our knowledge, only one study has been conducted among PD patients linking Vit B12 role, the methylation status and the neurodegenerative markers of PD including αSN (Obeid et al. 2009). In the study design, they tested serum concentrations of Vit B12, B6, SAM, Amyloid Beta_1-41_, Hcy and methylmalonic acid (MMA) among 87 PD patients. The results of the study did not suggest direct correlations between Vit B12 serum levels and αSN platelet levels.

##### Vit B12 and PD genetics

Growing evidence is pinpointing new facets of B12 in neurodegenerative diseases, such as AD and PD, as a gene and epigenetics regulator (El-Mezayen et al. 2022). In relation with PD, Vit B12 is considered as a natural Leucine-Rich Repeat Kinase 2 (LRRK2) kinase inhibitor. In fact, LRRK2 gene mutation (G2019S mutation) is known to cause the majority of autosomal dominant familial forms of PD and some of the sporadic cases (Rui et al. 2018). Such mutation induces a hyperactivity of the LRRK2 kinase, known to be highly neurotoxic. Unlike previously tested LRRK2 kinase inhibitors with significant side effects, Vit B12, derivative AdoCobalamin (AdoCb) specifically, exhibits a neuroprotective effect by regulating the activity of LRRK2 kinase (Schaffner et al. 2019) and has been shown to be effective in preventing neurotoxicity in cultured rodent neurons and able to mitigate dopaminergic deficit in these models.

Being a potential potent inhibitor of LRRK2 kinase activity with low risk for off-target side effects, Vit B12 supplementation in patients with PD (PwPD) seems appealing and encourages future clinical trials to investigate this potent effect as such evidence remains restrained to animal models (Green and Christine 2019) (Fig. 3).

##### Vit B12 and homocysteine

Vit B12 deficiency is linked to HHcy-induced-neurotoxicity. As shown in Fig. 2, HHcy is a surrogate marker of B12 deficiency. While there is a link of Hcy with neurodegeneration (Bonetti et al. 2016; Mattson and Shea 2003; Obeid and Herrmann 2006), little attention has been given to depict the mechanisms of how Hcy may impact on the neuropathological process in PD in particular. Recent evidence emerging from in-vitro studies, animal models and PD patients studies are starting to shed light on this matter (Al-Kuraishy et al. 2023).

HHcy aggravates the degeneration of dopaminergic neurons. Since Hcy can mimic the action of CNS excitatory neurotransmitters (McCully 2009), it produces an excess of intracellular influx of calcium which activates cellular apoptosis causing DNA cleavage and fragmentation (McCully 2009; Boldyrev 2009; Tyagi et al. 2005). Excitotoxicity, along with the increase of oxidative stress, embodies the major mechanisms of Hcy-induced neurodegeneration.

Furthermore, elevated Hcy can cause mitochondrial dysfunction, facilitates pathological aggregation of proteins such as SN and induces a toxic effect on dopaminergic neurons (Kumar et al. 2022; Bonetti et al. 2016). A recent study conducted on mice models of PD established that the N-homocysteinylation of α-SN enhances its aggregation potential and increases its neurotoxicity and that higher Hcy levels correlated with more severe α-synuclein deposition within mouse brains (Zhou et al. 2023). Finally, Hcy is responsible of neuro-inflammation in PwPD (Grotemeyer et al. 2022; Singh et al. 2020) **(**Fig. 3**)**.

### Dopamine and VIT B12: what’s the link?

The incidence of Vit B12 deficiency increases with advancing age and it is more prevalent among the LDopa treated subjects (Zhao et al. 2019). Many reports have found that total LDopa daily dose negatively correlated with Vit B12 levels (Romagnolo et al. 2019; Zis et al. 2017). PD patients who received long-term treatment with LDopa also exhibited notably lower levels of serum Vit B12 and folate when compared to age-matched controls (Ceravolo et al. 2013; Lizárraga and Lang 2022; Mancini et al. 2014). Both oral-treated and LCIG patients could exhibit alterations in serum B12 levels, HHcy and increased MMA (Romagnolo et al. 2019; Mancini et al. 2014; Comi et al. 2014; Rispoli et al. 2017; Toth et al. 2010; Triantafyllou et al. 2007).

Potential interactions between dopamine and B-group vitamins have been particularly investigated regarding their link with the greater incidence of peripheral neuropathy (PN) among PwPD (Ceravolo et al. 2013; Mancini et al. 2014). This yielded to conflicting results, raising questions about whether PN could be a direct consequence of their interaction or an additional systemic feature of PD on which B12 deficiency can act as a negative predictive factor (Comi et al. 2014).

Prior to the LDopa/carbidopa intestinal gel (LCIG) era, subclinical or clinical mild PN was observed in up to 50% of PD patients receiving oral LDopa. The presence of PN in PD subjects has been found to be associated with various factors in two large studies, including the total dosage of LDopa, low serum Vit B12 levels, and elevated Hcy and MMA levels (Ceravolo et al. 2013; Mancini et al. 2014). Subclinical signs of peripheral PN could be found in oral LDopa treated patients during the course of the disease, and low Vit B12 levels may facilitate the development of a clinically manifest PN.

However, several research papers did not establish significant correlations between mean LDopa daily dose, Vit B12 levels and PD-related PN (Corrà et al. 2023; Lamberti et al. 2005; Rajabally and Martey 2013). Some of these reports are limited by the small sample size, the small amount of LDopa administered as well as a large variability of age and disease duration in the populations of study (Corrà et al. 2023; Lamberti et al. 2005). In line with these findings, a study group found phosphorylated αSN deposits in proximal peripheral nerves of PD patients with small nerve fiber PN compared to atypical parkinsonism and healthy controls, with no significant differences between LDopa exposure or Vit B12 deficiency (Donadio et al. 2014).

#### Levodopa and cobalamin-related neurotoxic metabolites and benefits of COMT inhibitors

The breakdown of LDopa through COMT requires methyl group transfer, leading to the formation of the stable compound 3-O-MethylDopa (Müller and Riederer 2023). When high doses of LDopa are administered chronically, there is an increased demand for SAM as a methyl-group donor (Uncini et al. 2015). This could lead to Vit B12 depletion and transforms SAM into S-adenosyl-homocysteine and then into Hcy, the latter serving as a marker for methylation capacity (Müller and Riederer 2023). Consequently, Vit B12 levels may decrease in PwPD due to heightened methylation requirements associated with LDopa therapy and alter myelin synthesis throughout carbohydrate and fat metabolism contributing to the development of NP (Uncini et al. 2015). COMT inhibitors (COMT-I) can rebalance this abnormal metabolic loop by suppressing the overproduction of SAH by COMT after LDopa administration and lowering Hcy levels (Cossu et al. 2016; Zoccolella et al. 2005) (Fig. 2). Recent evidence indicates that Opicapone's greater bioavailability of LDopa likely accounts for the absence of a significant decrease in Hcy levels in chronic LDopa users, in contrast to the reduction observed with Entacapone. Nevertheless, both medications effectively prevent its elevation, highlighting HHcy as a reliable marker of methylation dysfunction (Müller et al. 2022).

HHcy generally correlated with cumulative LDopa dose without significant association with vitamin levels (Mancini et al. 2014; Comi et al. 2014; Mathukumalli et al. 2020; Miller et al. 2003; Müller 2008; Müller and Kuhn 2009). Consequently, it has been proposed that LDopa itself could drive the increase of Hcy, even without Vit B12 deficiency (Miller et al. 2003). HHcy is also considered as an independent factor of peripheral nerve damage (Uncini et al. 2015; Merola et al. 2016). In fact, electrophysiological studies have linked elevated Hcy levels in LDopa-treated patients to axonal loss in the sural nerve and a tendency towards weight loss among these patients (Cossu et al. 2016; Zoccolella et al. 2005; Kim et al. 2023). Using a combined COMT-I/LDopa treatment in PD patients has also shown to be effective in lowering Hcy levels and PN incidence when compared to oral LDopa alone (Andréasson et al. 2017). A single-nucleotide polymorphism (sA158G rs4680) in the COMT gene also led to a greater risk of LDopa-related PN in PwPD through low enzymatic activity (Toth et al. 2010).

Measuring MMA levels improves diagnostic sensitivity and specificity for cobalamin deficiency not only because Hcy elevation can occur in various conditions, but also because approximately 50% of cobalamin-deficient patients may have normal serum cobalamin levels leading to under-diagnosis (Romagnolo et al. 2019; Rajabally and Martey 2013; Taher et al. 2022; Toth et al. 2010). MMA could serve like an early biomarker of functional B12 deficiency. It's also worth noting that almost all patients with PD-associated PN have shown elevated MMA levels. The cumulative lifetime intake of LDopa and serum MMA levels might correlate with the extent of PN progression and severity (Müller 2008).

Does the route of administration matter?

To date, whether the route of LDopa administration impacts Vit B12 profile and PN risk or not remains a controversial matter (Romagnolo et al. 2019; Jugel et al. 2013). The global adoption of LCIG therapy, led to some reports of potential complications related to peripheral nerve toxicity and abnormal vitamin metabolism (Taher et al. 2022; Antonini et al. 2007; Loens et al. 2017; Santos-García et al. 2011). Recent global registry based on post marketing analysis of LDopa infusion use in real life population pointed out that discontinuation rates due to the occurrence of PN was only noted in two cases (Chaudhuri et al. 2023). Yet, it's important to underline that the study did not include a blood assessment of B12 or Hcy levels, nor involved nerve conduction studies. In the study conducted by Mancini et al., PN incidence was higher among patients treated with LCIG and/or oral LDopa in comparison with alternative dopaminergic treatments: emphasizing further the link between LDopa and PN. Simultaneously, a prospective investigation proposed that LCIG patients exhibited similar PN patterns compared to those seen in oral LDopa patients (Loens et al. 2017; Lehnerer et al. 2014).

**Fig. 4:**
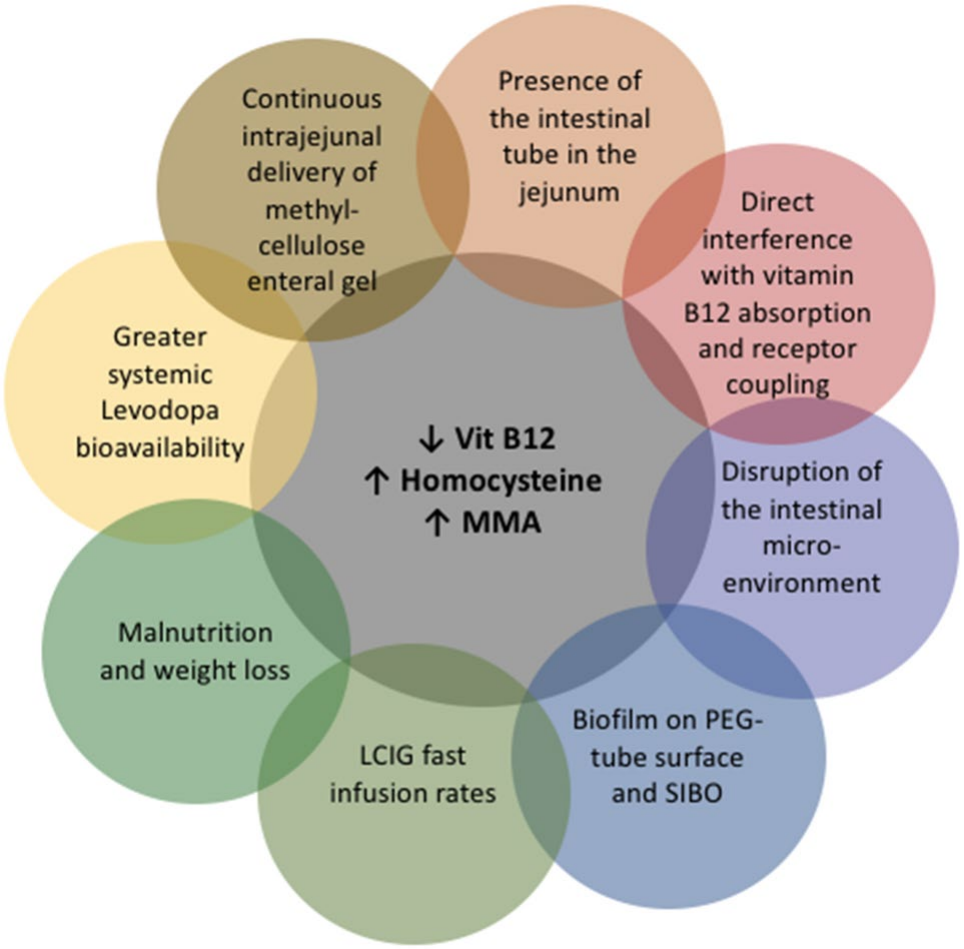
Potential causes of vitamin B12 deficiency in PD patients undergoing LCIG therapy. *MMA:* methylmalonic acid, *LCIG* levodopa–carbidopa intestinal gel; *PEG* percutaneous endoscopic gastrostomy, *SIBO* small intestinal bacterial overgrowth

A robust link between higher LCIG doses and reduced B12 levels in PwPD with PN has been pointed out with beneficial effect of vitamin B supplementation. Several mechanisms have been implicated leading to Vit B12 deficiency, HHcy and increased levels of MMA **(**Fig. 4**)** (Mancini et al. 2014; Merola et al. 2016). As a matter of fact, a large amount of LDopa is delivered in that specific part of the small intestine in a methylcellulose gel form and this might hinder vitamin absorption interfering with its receptor coupling (Lehnerer et al. 2014; Aasheim et al. 2008). This phenomenon appeared to be dose-dependent and influenced by the infusion rate. Faster infusion rates have been also associated with weight loss majoring, as a consequence, the risk factor for PN (Merola et al. 2016; Klostermann et al. 2012; Pauls et al. 2021). Thus, weight loss seems to be an additional factor linked to Vit B12 deficiency and PN. In the DUOGLOBE study previously mentioned (Chaudhuri et al. 2023), approximately 20% of the participants witnessed a reduction in weight by 7% or greater but most of the individuals retained their initial BMI ultimately. Lastly, intrajejunal continuous infusion provides a greater systemic bioavailability of LDopa compared to the same equivalent oral daily dose. This could interfere more intensively with Vit B12 intra-cellular metabolism nullifying the "physiological metabolic rest" of these components (Merola et al. 2016; Santos-García et al. 2012).

Regarding subcutaneous administration of LDopa, there is currently not enough data regarding its interaction with Vit B12 neither regarding potential sides effects such as PN. Thus, it is mandatory for post marketing surveillance to tackle this issue.

### Clinical peculiarities of VIT B12 deficiency in PD

#### Motor clinical correlates of Vit B12 deficiency in PD

To our knowledge, data to address the impact of Vit B12 deficiency on the motor aspect of PD is mostly lacking. In one study, authors pointed out that PwPD presenting with Vit B12 deficiency have significant white matter lesions which are thought to be both of microvascular and demyelinating origins. These patients presented with postural instability gait disturbance (PIGD) PD phenotype (Christine et al. 2018). Such finding was ultimately confirmed by a later study conducted by the same team, investigating the clinical correlates of Vit B12 levels in the cerebrospinal fluid (CSF). It further highlighted that lower CSF levels of Vit B12 correlated with higher UPDRS ‘walking’ item (Christine et al. 2020).

More rapid motor progression has also been associated to Vit B12 deficiency and HHcy as highlighted in the study conducted by M. C. Bakeberg et al. encompassing 205 PwPD. The authors demonstrated that male PwPD presenting with HHcy had significantly lower levels of Vit B12 and more rapid motor progression. In fact, they noted an increase of 0.77 points on the UPDRS part III for each additional 1 μmol/L of serum Hcy (Table 1) (Bakeberg et al. 2019**).**

**Table 1:** Clinical correlates of vitamin B12 and homocysteine levels in terms of motor and non-motor phenotypes of PD as established in literature

	Phenotype	Reference of the article (Year)	Cohort	Clinical features	Vit B12 status correlation	Homocysteine status correlation
Motor	PIGD phenotype	Christine et al. (2018)	680 untreated PwPD	Postural instability and falls among early PD	**↓ (serum)**	*NC*
		Christine et al. (2020)	581 untreated PwPD	Higher UPDRS-III ‘walking’ item	**↓ (CSF)**	*NC*
	Rapid Motor progression	Bakeberg et al. (2019)	205 PwPD and 78 HC	Greater motor deterioration of UPDRS-III score in male PD patients	**↓ (serum)**	**↑ (serum)**
Non-motor	Cognitive subtype	Zhong et al. (2022)	99 PwPD: 34 with MH (PD-MH), and 65 without MH	Among PD-MH group: Executive dysfunction Mood disturbances Gastrointestinal dysfunction Altered sleep quality	*NC*	**↑ (serum)**
		Christine et al. (2018)	680 untreated PwPD	Lower MMSE score	*NC*	**↑ (serum)**
		Bakeberg et al. (2019)	205 PwPD and 78 HC	Poorer cognitive performance in female PD patients based on MMSE score	*NC*	**↑ (serum)**

The bold indicates are abnormal findings

*PIGD* Postural instability gait disturbance, *PwPD* Patients with Parkinson’s disease, *HC* Healthy controls, *MH* Minor hallucinations, *UPDRS-III* Unified Parkinson’s Disease Rating Scale part III, *MMSE* Mini-mental status examination, *NC* No statistically significant correlation

#### Non-motor clinical correlates of Vit B12 deficiency in PD

More extensive research investigated the non-motor aspects of PD directly linked to HHcy rather than focusing of Vit B12 deficiency itself. However, taking into consideration that Vit B12 deficiency can induce the elevation of Hcy levels, it seems plausible to consider the non-motor aspects related to Hcy (Shipton and Thachil 2015).

Regarding cognition, one meta-analysis pointed out that PD patients with cognitive impairment were more prone to having higher Hcy levels, lower folate and lower Vit B12 levels (Xie et al. 2017). Increased Hcy levels were linked to poorer cognitive performance in female PwPD (Bakeberg et al. 2019). In a recent cross-sectional study including 99 PwPD, thirty-four patients presented with minor hallucinations which correlated with HHcy (Zhong et al. 2022). Christine et al. also pointed out that baseline elevated Hcy among untreated de novo PwPD was associated with lower baseline MMSE as well as greater cognitive decline during disease’s progression (Christine et al. 2018). However, cognitive dysfunction was linked to Hcy and did not correlate with Vit B12 levels in any of these studies (Table 1).

Another study has unveiled that LDopa-treated PwPD who experienced cognitive impairment as measured through MMSE, had substantially lower serum Vit B12 levels in comparison to both LDopa-treated PD patients without cognitive deficits and the control group (Al Amin and Gupta 2023). A recent meta-analysis also showed HHcy in LDopa-treated patients when compared to those not receiving it which lines with the idea that the administration of LDopa contributes to HHcy in PD. The presence of HHcy also correlated with lower cognitive performances in PwPD compared to cognitively unimpaired patients. Nonetheless, the same work also highlighted that the entire cohort of PD patients displayed elevated Hcy levels when compared to the control group, regardless of the received treatment (Schaffner et al. 2019). These findings suggest that increased Hcy concentrations in PD patients may arise from multiple factors beyond LDopa itself.

### VIT B12 supplementation impact on PD: guidelines and recommendations

#### B12 supplementation: Pathogenesis, motor and non-motor outcomes in PD

As pointed out in the first sections of this article, Vit B12 does significantly impact PD pathogenesis. Data from animal PD models and in-vitro experiments are suggestive of the neuroprotective effect of Vit B12. Since the list of contraindications of Vit B12 is really restrained to Leber optic neuropathy as it may aggravate it and to advanced renal failure since the injections may include aluminum in its components, we may suggest to consider systematically supplementing PD patients with B12 in the absence of contraindications or potential adverse effects (McCarter et al. 2020b). Patients with LRRK2 gene mutation can be great candidates for Vit B12 supplementation (Obeid and Herrmann 2006). Yet, in order to solidify such suggestion, we do require case–control double blinded studies among PwPD in different stages of the disease’s progression.

Yet, the role of Vit B12 supplementation in controlling motor progression of PwPD remains controversial. Until now, there is no concrete indication for Vit B12 therapy in order to moderate the motor burden of PD. However, since PwPD with PN seem to have worse gait and balance scores due to the peripheral involvement of both small and large fibers (Corrà et al. 2023), Vit B12 supplement can be provided to them in order to improve at least one of the many aggravating factors of balance in PD. HHcy is considered a cardiovascular risk factor and could cause microvascular lesions within the CNS leading to the accumulation of motor disability in PwPD. Thus, it seems only plausible to act actively by providing vitamin supplementation in patients aiming at lowering Hcy levels, especially in older PwPD.

Regarding non-motor symptoms in PD, Vit B12 has been individually linked to cognition and is used as a therapy for Vit B12 related cognitive impairment (McCarter et al. 2020b). Yet, Vit B12 deficiency and HHcy are both catalysts for a singular non-motor PD phenotype characterized with cognitive dysfunction. Higher baseline Vit B12 has been associated with lower risk of developing dementia among PwPD (Müller et al. 2013). In this latter study, a cut-off value of Vit B12 has been established (> 587 ng/L) to reduce the risk of developing dementia with reasonable sensitivity and specificity at 5 and 10 years from PD diagnosis. Thus, we recommend to regularly assess cognitive function among PwPD, test Vit B12 serum levels along with Hcy and MMA levels.

#### B12 supplementation, Levodopa and LCIG: a delicate matter

**Fig. 5:**
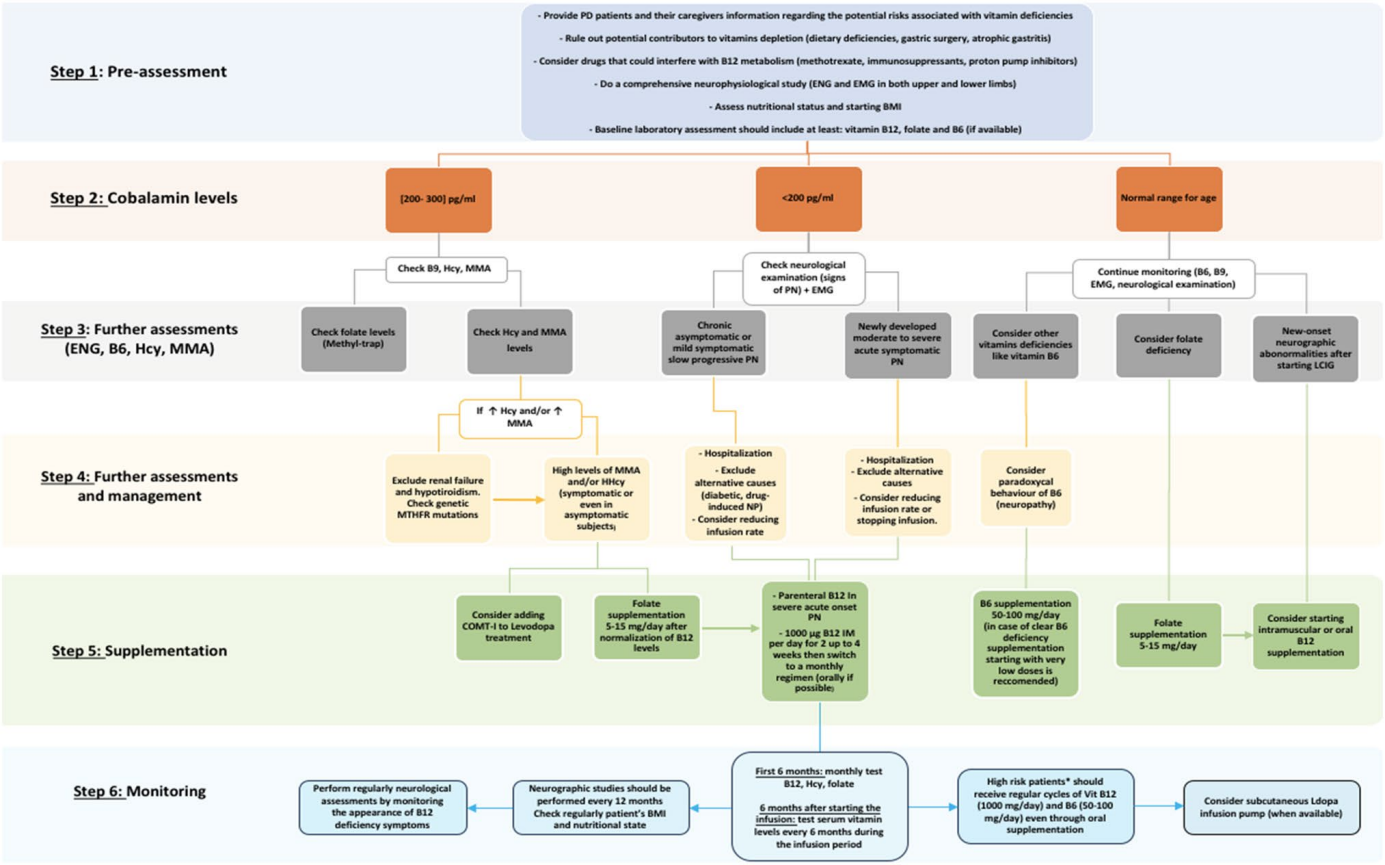
Flowchart of the proposed guidelines for the management of LCIG–treated patients. Step 1 (pre-assessment) is followed by the evaluation of Vit B12 serum levels as a main criterion to determine the following approach (Step 2). In step 3, based on Vit B12 status, supplementary clinical, laboratory and neurophysiological tests are indicated. Step 4 dictates how to approach HHcy or elevated MMA levels and neuropathy according to its severity. Step 5 is the supplementation protocole. Finally, step 6 pinpoints the monitoring approach. *PD* Parkinson disease, *HHcy* Hyperhomocisteinemia, *ENG* Electroneurography, *EMG* Electromyography, *BMI* Body-mass index, *pg/ml* picograms per milliliter, *MMA* Methylmalonic acid, *PN* peripheral neuropathy, *LCIG* levodopa–carbidopa intestinal gel, *MTHFR* Methylenetetrahydrofolatereductase, *COMT-I* Catechol-O-methyltransferase inhibitor, *IM* intramuscular, *μg* micrograms. *High risk patients = (advanced PD, diabetic patients, pre-existing PN)

As detailed in Fig. 5, we strongly recommend that the pre-assessment for PwPD who are candidates for LCIG includes a comprehensive neurophysiological study to unmask potential subclinical or clinical PN. Ruling out other potential causes of PNs and assessing the baseline nutritional status and the BMI is also advisable. Potential drugs and medication that could alter vitamins absorption along with personal history of gastric surgery or chronic gastritis should be considered as additional risk factors of Vit B12 deficiency (Uncini et al. 2015). First level laboratory assessment should include vitamins B12 and folate serum and pyridoxine levels if possible. Nevertheless, it's essential to acknowledge the considerable inter-individual variability in B12 levels, complicating the establishment of a universal deficiency threshold due to a lack of standardization (Taher et al. 2022; Toth et al. 2008). Hence, it is prudent to measure also Hcy and MMA levels systematically. Laboratory tests should always be interpreted in the light of the patient’s personal history and ongoing medication since Hcy levels can increase in renal failure, hypothyroidism and in some genetic metabolic conditions (Romagnolo et al. 2019; Uncini et al. 2015; Müller et al. 2013). If neurographic abnormalities are detected in an asymptomatic subject and even with normal Vit B12 levels, we suggest starting B12 supplementation if LCIG infusion has to be started.

In view of a clear Vit B12 deficiency and/or HHcy and/or high MMA levels in a PwPD presetting with clinical signs of PN, a complete neurographic assessment and an extensive alternative-causes workup should be performed. Supplementation should be started immediately to minimize the risk of delayed and suboptimal recovery from axonal degeneration. In this case, decision to start LCIG should be carefully considered in patients already presenting with symptomatic PN based on clinical judgement and the patient’s real clinical benefit-sharing (Santos-García et al. 2012). In case of high-risk patients or new-onset subclinical neurographic changes, starting slowly or lowering the speed and the dose of infusion respectively, might help avoiding the risk of progression of the PN and even improve the PN (Rispoli et al. 2017).

However, Vit B12 supplementation as a preventive treatment remains questionable. For some instance, PN can even manifest in spite of having normal vitamin levels and despite receiving an appropriate Vit B12 replacement therapy (Uncini et al. 2015; Taher et al. 2022; Müller et al. 2013). Most non disease-specific guidelines suggest a daily or alternate day dose of 1000 μg of intramuscularly B12 for 2 weeks and then a transition to a monthly regimen (Kramarz et al. 2023). However, high risk patients (advanced PD, diabetic patients, subclinical PN) who are considered as more prone to developing symptomatic PN should receive regular injections of Vit B12 and B6 (Uncini et al. 2015; Taher et al. 2022; Loens et al. 2017).

Besides Vit B12 supplementation, additional measures could be considered such as Vit B9 supplementation and the use of COMT-I. Folate levels should only be tested if cobalamin levels are normal. An unintended consequence of supplementing folate deficiency without first assessing B12 levels can lead to the apparent rise of Vit B12 levels (“methyl-trap”) while a functional Vit B12 persists maintain the neurological issues (Cossu et al. 2016; Taher et al. 2022). As for the use of COMT-I, has been linked to a reduced incidence of LDopa-associated PN regardless of disease duration and severity, likely due to the positive impact on Hcy production (Phokaewvarangkul et al. 2023): Nevertheless, literature on COMT-I is controversial and other studies have not yielded similar beneficial effects (Uncini et al. 2015). Regarding novel therapies, data regarding Opicapone and intestinal infusion of LECIGON effects on Vit B12 and Hcy levels is missing (Rispoli et al. 2017) and could represent a significant aspect to investigate in future studies.

In terms of monitoring, it is advisable to conduct monthly tests for B12, Hcy and MMA during the initial 6 months, followed by tests every 3–6 months in LCIG patients (Jia et al. 2019). Neurographic study should be performed every 6–12 months during LCIG infusion. Patient’s weight and BMI changes should always be kept under control and compared to the baseline values since a rapid decrease in BMI could be considered as an indicator of malnutrition and predisposition to LCIG-related complications.

### Limitations

This review is subject to several limitations inherent in the included studies as great evidence is derived from animal and in-vitro models of PD. Variability in Vit B12 assessment methods, PD diagnostic criteria, and study populations may contribute to heterogeneity in the results. Additionally, most studies were observational in nature, limiting the ability to establish causality. The potential for publication bias and the exclusion of non-English articles may also impact the comprehensiveness of the findings.

## Conclusions

Compelling evidence has been provided pinpointing that Vit B12 effectively hinders the formation of αSN fibrils and reduces neuronal cytotoxicity. Such conclusion was mainly established based on animal models of PD and in-vitro experiments. Furthermore, the question whether Vit B12 should be considered as symptomatic treatment in PD or as a disease modifying therapy remains triggering and unresolved. Thus, it is essential to approach such hypothesis with caution and aim to demonstrate it in case–control studies including PwPD.

As for clinical correlates, Vit B12 deficiency and HHcy do predict more rapid motor progression and cognitive dysfunction among PwPD and thus modulate PD clinical phenotypic variability. We highlighted that PN is an underestimated problem among PwPD, especially among those receiving LCIG infusion. It remains crucial to regularly warrant a close monitoring of Vit B12, Hcy and MMA as well as the nutritional status of PwPD in general. We provided a guideline detailing the key aspects of managing LCIG-treated PwPD. In this context, the subcutaneous LDopa infusion pump could offer an intermediate approach between the two conventional administration routes of LDopa, potentially mitigating some side effects.

Finally, while Vit B12 supplementation has practical implications in neurological diseases and seems to yield benefits in PD, the dearth for comprehensive understanding surrounding its CNS bioavailability and mechanisms of action presents an obstacle to elucidate among PD patients. As we move forward, it becomes increasingly evident that further interventional studies are essential to provide evidence-based valuable insights and recommendations regarding the monitoring of Vit B12 deficiency and its biomarkers and to establish accurate guidelines for Vit B12 supplementation in PD.
